# Probiotics Supplements Reduce ER Stress and Gut Inflammation Associated with Gliadin Intake in a Mouse Model of Gluten Sensitivity

**DOI:** 10.3390/nu13041221

**Published:** 2021-04-07

**Authors:** Eleonora Ferrari, Romina Monzani, Valentina Saverio, Mara Gagliardi, Elżbieta Pańczyszyn, Valeria Raia, Valeria Rachela Villella, Gianni Bona, Marco Pane, Angela Amoruso, Marco Corazzari

**Affiliations:** 1 Department of Health Science, University of Piemonte Orientale, 28100 Novara, Italy; eleonora.ferrari@uniupo.it (E.F.); romina.monzani@uniupo.it (R.M.); valentina.saverio@uniupo.it (V.S.); mara.gagliardi@uniupo.it (M.G.); elzbieta.panczyszyn@uniupo.it (E.P.); 2 Center for Translational Research on Autoimmune and Allergic Disease (CAAD), University of Piemonte Orientale, 28100 Novara, Italy; 3 Regional Cystic Fibrosis Center, Pediatric Unit, Department of Translational Medical Sciences, Federico II University Naples, 80134 Naples, Italy; raia@unina.it; 4 European Institute for Research in Cystic Fibrosis (IERFC-Onlus), San Raffaele Scientific Institute, 20132 Milan, Italy; valeria.villella@gmail.com; 5 Division of Pediatrics, Department of Health Science, University of Piemonte Orientale, 28100 Novara, Italy; gianni.bona@maggioreosp.novara.it; 6 Probiotical Research Srl, 28100 Novara, Italy; m.pane@probiotical.com (M.P.); a.amoruso@mofinalce.it (A.A.); 7 Interdisciplinary Research Center of Autoimmune Diseases (IRCAD), University of Piemonte Orientale, 28100 Novara, Italy

**Keywords:** CD, UPR, TG2, CFTR, probiotics

## Abstract

Exposure to gluten, a protein present in wheat rye and barley, is the major inducer for human Celiac Disease (CD), a chronic autoimmune enteropathy. CD occurs in about 1% worldwide population, in genetically predisposed individuals bearing human leukocyte antigen (HLA) DQ2/DQ8. Gut epithelial cell stress and the innate immune activation are responsible for the breaking oral tolerance to gliadin, a gluten component. To date, the only treatment available for CD is a long-term gluten-free diet. Several studies have shown that an altered composition of the intestinal microbiota (dysbiosis) could play a key role in the pathogenesis of CD through the modulation of intestinal permeability and the regulation of the immune system. Here, we show that gliadin induces a chronic endoplasmic reticulum (ER) stress condition in the small intestine of a gluten-sensitive mouse model and that the coadministration of probiotics efficiently attenuates both the unfolded protein response (UPR) and gut inflammation. Moreover, the composition of probiotics formulations might differ in their activity at molecular level, especially toward the three axes of the UPR. Therefore, probiotics administration might potentially represent a new valuable strategy to treat gluten-sensitive patients, such as those affected by CD.

## 1. Introduction

Gluten exposure, a protein present in wheat, rye, and barley, is the major inducer for human Celiac Disease (CD), a chronic autoimmune enteropathy. CD occurs in about 1% of worldwide population, in genetically predisposed individuals carrying human leukocyte antigen (HLA) DQ2/DQ8 [1]. In CD patients, gliadin, a gluten component, binds the chemokine CXC motif receptor 3 (CXCR3), thus promoting the release of Zonulin, resulting in the disassembly of tight junctions, with gliadin crossing the epithelial barrier. Next, through tissue transglutaminase 2 (TG2) activity, deamidated gliadin binds the HLA-DQ2/8 molecules on antigen-presenting cells (APCs) [2,3] and then presents on CD4^+^T cells, resulting in their activation and migration to the small intestinal lamina propria [4]. Once in the lamina propria, activated CD4^+^T cells proliferate and start to produce proinflammatory cytokines, such as IFNγ and metalloproteases, and stimulate the production of growth factors by stromal cells, which induces cryptal hyperplasia and villous atrophy due to intestinal epithelial cells death induced by intraepithelial lymphocytes (IELs) [5]. Recent studies have indicated a new actor playing a role in the pathogenesis of CD, the cystic fibrosis transmembrane conductance regulator (CFTR) [6], linking CD to Cystic Fibrosis (CF). CF is the most common genetic lethal disease in the Caucasian population and is caused by loss-of-function mutations of the *cftr* gene. Although CFTR was originally identified as a cAMP-activated transmembrane anion channel mediating the transport of Cl-/HCO_3_^−^ across the epithelia, it is now also recognized as a hub protein regulating and orchestrating a complex protein network in epithelial cells. Loss-of-function mutations of CFTR cause an increased reactive oxygen species (ROS) production, the activation of TG2, the inhibition of autophagy, and a defective bacterial killing [7,8,9]. Moreover, active TG2 leads to NF-*κ*B activation, causing an increased level of proinflammatory cytokines such as IL-15, IL-17A, and IL-21, the main cytokines involved in CD pathogenesis. Importantly, gliadin binds to the NBD1 domain of CFTR, thus inhibiting its gating functions and impairing autophagy and proteostasis [6,10]. To close this vicious circle, inhibited CFTR sustains, in turn, the activity of TG2, which enhances the amount of deamidated gliadin, finally raising its antigenicity [10].

Importantly, the abovementioned activation of TG2 seems to rely on gliadin-stimulated intracellular calcium mobilization from the endoplasmic reticulum (ER), a condition potentially resulting in imbalanced ER homeostasis known as ER stress [11], as evidenced in vitro by Caputo and colleagues [12]. Notably, perturbed ER homeostasis seems to be a feature of inflammatory intestinal disorders such as inflammatory bowel disease (IBD) [13], although its role is still under investigation.

Actually, the only effective treatment for CD is a lifelong gluten-free diet, although many patients have many difficulties adhering to this restriction diet throughout their life. However, new therapeutic approaches have been suggested which include Zonulin receptor inhibitors, engineered gluten-free grain, TG2 inhibitors, and probiotics in addition to the diet [14,15,16]. The latter intervention is focused on buffering the gut microbiota dysregulation, which has been described to influence the CD pathogenesis through the modulation of intestinal permeability, regulation of the immune system, and modulation of the digestion of gluten-generating toxic and tolerogenic peptides [17]. Indeed, gut microbiota dysbiosis has been associated to the development and progression of several chronic gut diseases such as IBD [18], Colitis [19], and CD [20]. Interestingly, dysbiosis often persists unexpectedly in some CD patients despite the gluten-free diet, with this condition potentially and surprisingly being induced by this restricted diet.

In the present study, using our well-characterized mouse model of gluten sensitivity [6], we tested the hypothesis that the administration of probiotic’ supplements relieve the intestinal dysfunctions due to gluten intake observed in CD patients.

## 2. Materials and Methods

### 2.1. Mice and Treatments

Balb/c mice were obtained from Charles River (Calco). A total of 36 eight-week-old, third-generation, gluten-free mice (Mucedola) were randomly divided into 6 groups (G1–G6), composed of 6 mice/group. G1 was challenged with a gluten-free diet for the duration of the experiment. G2−G4 were challenged with oral gavage with gliadin (Sigma; 5 mg/daily for 1 week, then 5 mg/daily thrice a week for 3 weeks) for 4 weeks [6,21]. At the end of the fourth week, mice from G2−G6 were challenged with gliadin alone (G2) or in combination with P1 (G3) or P2 (G4), or with P1 (G5) or P2 (G6) alone, every day via oral gavage for another 2 weeks (P1: 5.7 × 10^5^ ± 2.0 × 10^5^/day; P2: 8.5 × 10^5^ ± 2.0 × 10^5^/day; equivalent doses in mice, calculated on the basis of those recommended for humans [http://www.salute.gov.it/imgs/C_17_pubblicazioni_1016_ulterioriallegati_ulterioreallegato_0_alleg.pdf; accessed on 1 December 2020]). A schematic representation of treatments is reported at the end of the paragraph. At the end of the last daily treatment (7 weeks), all mice were sacrificed, and the intestine and blood were collected and used for the analysis described below.

All procedures were approved by the local Ethics Committee for Animal Welfare (IACUC No 849) and conformed to the European Community regulations for animal use in research (2010/63 UE). 


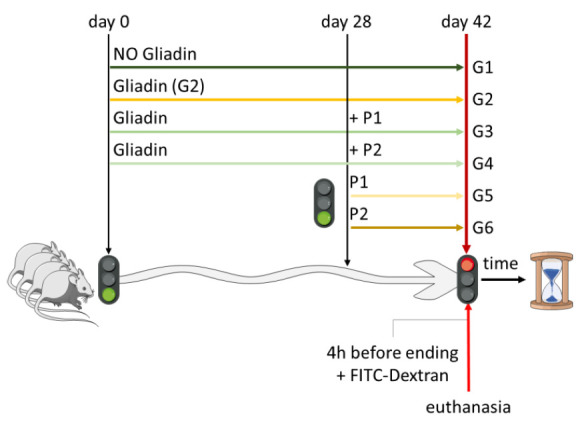


### 2.2. Cell Lines and Treatments

Human colon adenocarcinoma-derived Caco-2 cells were obtained from ATCC. Cells were maintained in T25 flasks in Dulbecco’s Modified Eagle Medium (DMEM) (Sigma) supplemented with 10% fetal bovine serum (FBS), 2 mM glutamine (Sigma), and 1% penicillin/streptomycin (Sigma). All experiments were performed in the absence of antibiotics. In a 6-well plate, 3 × 10^5^ cells/well were seeded and treated or untreated 3 h or 9 h (with or without refresh of the treatment every 3 h, as indicated) with pepsin-trypsin-gliadin (PT gliadin; 1 mg/mL) with or without 1 × 10^9^ bacterial cells of probiotic formulation 1 (P1) or 2 (P2). PT was prepared as previously described [22] with minor modifications. Briefly, 50 g of gliadin (Sigma) was dissolved in 500 mL 0.2 N HCl for 2 h at 37 °C with 1 g of pepsin (2.5 units/mg protein; Sigma). The resultant peptic digest was further digested by the addition of 1 g trypsin (Sigma) after the pH was adjusted to 7.4 using 2 M NaOH. The solution was stirred vigorously at 37 °C for 4 h, boiled to inactivate enzyme for 30 min, lyophilized, and then stored at −20 °C until used. PT gliadin was freshly resuspended in a sterile phosphate-buffered saline (PBS). A schematic representation of the experimental procedure is reported in corresponding figure.

### 2.3. Probiotics Formulations

Probiotics were supplied by PROBIOTICAL SpA, in lyophilized powder. The P1 formulation contained 2 strains of Bifidobacterium breve, the B632 (DSM 24706) and BR03 (DSM16604) at 2 × 10^9^ live cells (AFU)/g, while the P2 formulation contained the Lactobacillus plantarum LP14 (DSM 33401), *L. casei* subsp. paracasei LPC09 (DSM 24243) and the Lactobacillus rhamnosus LR04 (DSM 16605) at 3 × 10^9^ live cells (AFU)/g. The study materials were analyzed by Probiotical Research srl, Novara, Italy, via flow cytometry (ISO 19344:2015 IDF 232:2015) to confirm target cell count. P1 or P2 were resuspended in PBS and administrated as described.

### 2.4. Intestinal Permeability Assay

The fluorescein isothiocyanate-conjugated dextran (FITC-Dextran 4000; Sigma) was used to perform the intestinal permeability assay using 4 animals/group of Balb/c mice treated as described in the previous section. Briefly, FITC-Dextran was oral gavaged to the mice at a concentration of 44 mg/100 g body weight at 4 h prior to the euthanasia. At the end of treatments, mice were anesthetized, and blood was collected by cardiocentesis, heparinized, centrifuged 10 min at 12,000× *g*, and plasma was protected. Next, each plasma was diluted with an equal volume of PBS, pH 7.4, and a standard curve was obtained though serially diluted FITC-Dextran stock solution (0 ng/mL; 125 ng/mL; 250 ng/mL; 500 ng/mL; 1000 ng/mL; 2000 ng/mL; 4000 ng/mL; 8000 ng/mL). Then, 100 µL of each diluted plasma (in quadruplicate) was transferred to a 96-well microplate. Fluorescence (485 nm ex; 528 nm em) was evaluated using a SPARK Multimode Microplate Reader (TECAN), and the FITC-Dextran concentration was obtained using the standard curve interpolation.

### 2.5. Western Blotting Analysis

The whole small intestine lysates were obtained using the Cell Lytic buffer (Sigma) supplemented with a protease inhibitors cocktail (Sigma) plus phosphatases inhibitors (Na3VO4 1 mM; NaF 10 mM), resolved by electrophoresis through SDS-PAGE, and electroblotted onto nitrocellulose (Protran, Sigma) membranes. Membranes were incubated with indicated primary antibodies in 5% nonfat dry milk (Bio-Rad) in PBS plus 0.1% Tween20 overnight at 4 °C. Primary antibodies were: Anti-CFTR (clone M3A7 Abcam ab4067) 1:500, anti-TG2 (NeoMarkers) 1:750, and anti-βActin (Cell Signaling) 1:2000. Detection was achieved using horseradish peroxidase-conjugate secondary antibody (1:5000; Jackson ImmunoResearch; Cambridge, UK) and visualized with ECL plus (Amersham Biosciences; Amersham). Images were acquired using a ChemiDoc™ Touch Imaging System (Bio-Rad) and analyzed by Image Lab software (Bio-Rad), as previously described.

### 2.6. ELISA

IL-15, IL-17A, and INFγ were measured in small intestine lysates using the Mouse IL-15 DuoSet ELISA, the Mouse IL-17 Quantikine ELISA Kit, or the Mouse IFNγ Quantikine ELISA Kit (Bio-Techne), as recommended by the supplier. ODs (optical densities) were analyzed by a SPARK Multimode Microplate Reader (TECAN). Values were normalized to total protein concentration evaluated by Bradford analysis, as previously reported [10].

### 2.7. Hematoxylin/Eosin Staining

After surgical removal of the small intestine, samples were fixed in formalin buffer at room temperature, dehydrated, and embedded in paraffin. Then, 8 μm-thick sections were collected using a microtome (Leica). All sections were mounted on slides and stained with hematoxylin and eosin (Bio Optica). Images were acquired using a Nikon Eclipse Ci Microscope, a Plan APO 20X Objective, and the NIS-Elements Software (Nikon).

### 2.8. Quantitative RT-PCR

Trizol reagent (Invitrogen) was used to isolate total RNA, as indicated by the supplier. The AMV Reverse Transcriptase kit (Promega) was used to generate cDNA following the manufacturer’s recommendations. Quantitative polymerase chain reaction (PCR) was performed using the CFX96 thermocycler (Bio-Rad). Supplementary Table S1 shows the primers sequence for all amplicons, designed using the online IDT PrimerQuest Tool software (IDT, Integrated DNA Technologies Inc., Coralville, IA, USA; https://eu.idtdna.com/Primerquest/Home/Index, accessed on 14 February 2021). Results were normalized using mouse GAPDH or human L34 as internal control.

### 2.9. Statistical Analysis

All experiments were performed at least in triplicate and statistical analysis was performed using GraphPad Prism 6. The Student’s *t*-test was used to determine statistical significance. A *p*-value of equal to or less than 0.05 was considered significant. In each table, **** *p* < 0.0001; *** *p* < 0.001; ** *p* < 0.01; * *p* < 0.05.

## 3. Results

### 3.1. Probiotics Administration Inhibits Gliadin-Mediated TG2 Upregulation but Does Not Restore CFTR Physiological Expression

TG2 is a key player in CD, since anti-TG2 autoantibodies are commonly found in active CD affected patient’s sera. Recently, the ability of gluten derived peptides to bind CFTR has been described, resulting in protein destabilization and subsequent degradation [6]. CFTR impairment also results in TG2 expression upregulation and activation—although the molecular mechanism is still unclear—which promotes the TG2-mediated gliadin peptides deamidation. In turn, deamidation causes an increased binding affinity of deaminated peptides to the disease-predisposing human leukocyte antigen (HLA) DQ2 and DQ8 molecules, thus enabling a strong immune response contributing to the pathogenesis of celiac disease.

**Figure 1:**
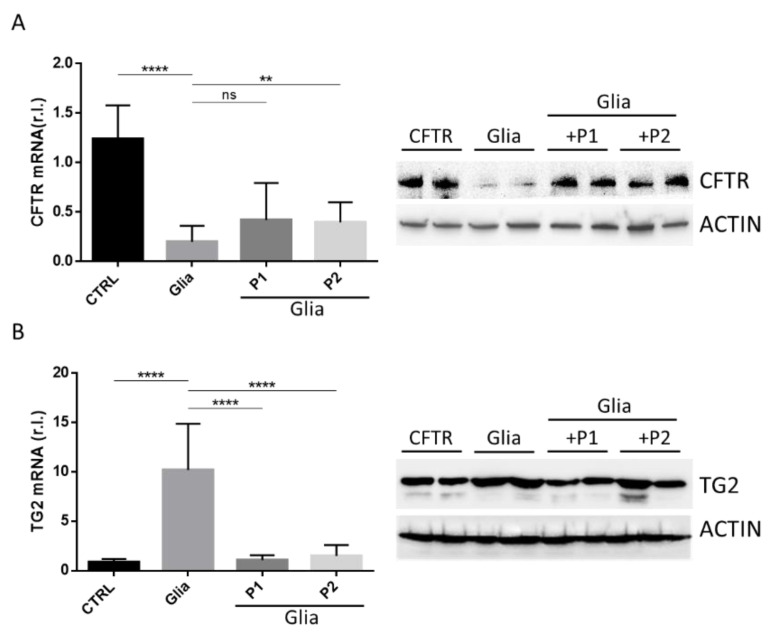
Tissue transglutaminase 2 (TG2) and cystic fibrosis transmembrane conductance (CFTR) modulation by probiotics administration in vivo. CFTR (**A**) and TG2 (**B**) expression levels were evaluated in the small intestine of Balb/c fed third-generation gluten-free mice, treated (Glia) or not treated (CTRL) with gliadin, in the presence or absence of P1 or P2, at both mRNA (left panels) and protein (right panels) levels. Histograms represent mean ± standard deviation (SD) of triplicate sample; **** *p* < 0.0001; ** *p* < 0.01; ns = not significant; β-actin was used as loading control in the immunoblots.

Therefore, we evaluated both CFTR and TG2 mRNA and protein levels in the small intestine of Balb/c gluten-sensitive mice exposed to gliadin for 4 weeks and to gliadin in presence or absence of P1 or P2 probiotics formulations for 2 more weeks. Data reported in Figure 1 show that gliadin exposure efficiently downregulated the expression of CFTR (A) and consistently elevated the expression of TG2 (B) at both mRNA and protein levels. Importantly, the concomitant administration of P1 or P2 efficiently inhibited the gliadin-induced TG2 upregulation at both mRNA (Figure 1B, right panel) and protein (Figure 1B, left panel) levels, suggesting the ability of these probiotics formulations to potentially reduce the damaging effect exerted by gliadin peptides.

Moreover, our data also show that the two probiotic formulations were able to restore, at least in part, the physiological protein levels of CFTR (Figure 1A, right panel), while no major effects were observed at mRNA levels (Figure 1A, left panel). Further studies are therefore required to better elucidate this apparent discrepancy. However, to our knowledge, this is the first evidence showing a gliadin-mediated TG2 and CFTR gene expression regulation.

Collectively, these data suggest that bacteria from the two formulations are unlikely to prevent the formation of the active gliadin peptides generated by digestion as some events downstream of gliadin, such as the downregulation of CFTR and upregulation of TG2 mRNAs, are unaffected by P1 and P2. The partially restored CFTR protein levels suggest that bacteria exert their protective effect, at least in part, downstream of the formation of proinflammatory peptides.

### 3.2. Dysregulated Intestinal Permeability Due to Gliadin Exposure Was Restored by Probiotics Administration

Altered intestinal permeability is one key feature of CD pathogenesis. In fact, although the ability of gliadin peptides to cross the intestinal epithelial barrier is prevented by the presence of an efficient Zonulae occludent established among epithelial cells under physiological conditions through means of thigh junctions (TJs) in genetically susceptible individuals, intestinal cells trigger the disassembly of TJs. Indeed, during the acute phase of CD, it has been reported that Zonulin, a protein involved in the regulation of intestinal permeability, is upregulated. Zonulin binding to its surface receptors leads to an opening of the tight junctions, resulting in an increase in intestinal permeability [23].

**Figure 2:**
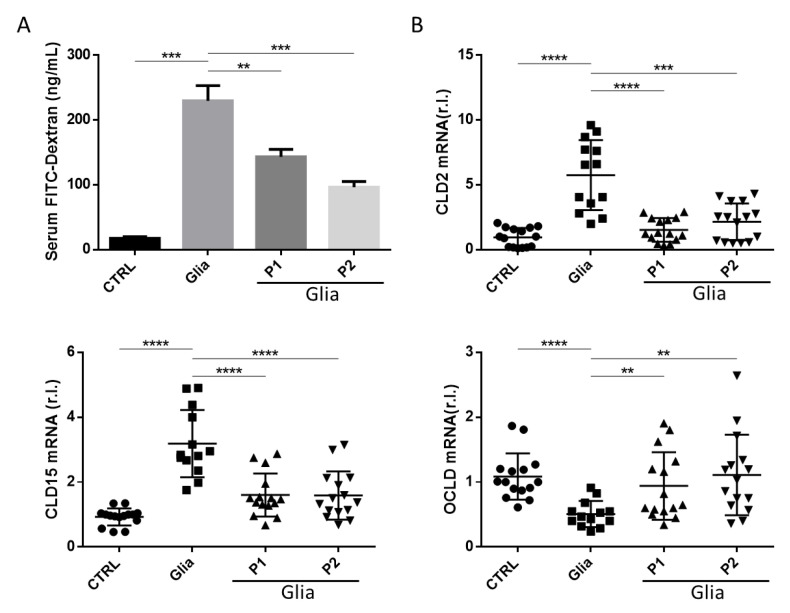
Gliadin-mediated intestinal permeability dysregulation is restored by probiotics administration. Plasma concentration of fluorescein isothiocyanate-conjugated (FITC) fluorescence was measured 4 h after mouse gavage of a single dose of FITC-Dextran (**A**). Quantification of plasma concentration from *n* = 2 mice per group. Mean ± SD of duplicate sample. (**B**) Claudin 2 (upper panel), Claudin 15 (bottom left panel), and Occludin (bottom right panel) expression levels were evaluated in the small intestine of third-generation Balb/c mice fed with gluten-free diet challenged with gliadin (Glia) in the presence or absence of P1 or P2 by quantitative real-time polymerase chain reaction (qRT-PCR). Mean ± SD of triplicate sample; **** *p* < 0.0001; *** *p* < 0.001; ** *p* < 0.01.

Therefore, we evaluated the intestinal permeability in our gliadin sensitive mice in vivo, exposing animals to gliadin for 6 weeks using The fluorescein isothiocyanate-conjugated dextran (FITC-Dextran) administrated as a single dose (through gavage; 44 mg/100 g body weight) at 4 h prior animal sacrifice [6,21]. Fluorescence (FITC), measured in the plasma of mice and reported in Figure 2A, shows a considerable increased permeability in mice exposed to gliadin compared to untreated control mice. These data are in line with the increased upregulation of both claudin 2 and 15 and downregulation of Occludin (three TJ components) observed in mice exposed to gliadin compared to controls and previously associated to abnormal intestinal permeability (Figure 2B) [24,25,26].

Importantly, the co-administration of P1 or P2 consistently inhibited the intestinal permeability abnormality mediated by the gliadin active peptides, as shown in Figure 2A. Moreover, these results were confirmed by the restored claudin 2 and 15 physiological expression in mice exposed to gliadin in presence of P1 or P2 (Figure 2B upper right and bottom left panels, respectively).

### 3.3. Gliadin-Mediated Small Intestinal Inflammation Was Buffered by Probiotics Administration

**Figure 3:**
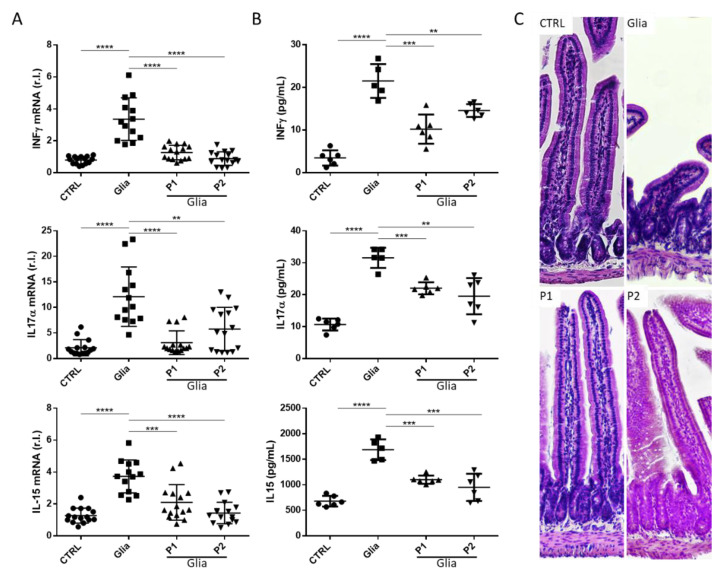
Anti-inflammatory activity of probiotics. IL-15, IL-17a, and INFγ mRNA (**A**) and protein (**B**) levels were evaluated in the small intestine tissue homogenate of Balb/c-fed third-generation gluten-free mice challenged with gliadin (GLIA) and P1 or P2 and compared to untreated control (CTRL) by qRT-PCR and ELISA, respectively. (**C**) H/E staining of small intestine from mice exposed (Glia), unexposed (CTRL) to gliadin alone or in combination with probiotic formulation 1 (P1) or 2 (P2). Images are representative of three independent experiments (magnification = 20×). Mean ± SD of triplicate sample **** *p* < 0.0001; *** *p* < 0.001; ** *p* < 0.01.

CD is a T-cell mediated disease, in which, gliadin-derived peptides activate T lymphocytes infiltrating the lamina propria, resulting in the production and release of pro-inflammatory cytokines. Indeed, we observed the upregulation of key pro-inflammatory cytokines, IFNγ–IL-15–IL-17A [6,21], in small intestine lysates of gliadin sensitive animals exposed 6 weeks to gliadin, compared to untreated controls (Figure 3A). Importantly, in line with data reported above, the presence of P1 or P2 consistently inhibited the upregulation of the pro-inflammatory genes and cytokine release (Figure 3A,B, respectively). However, although both probiotics formulations completely inhibited the gliadin-mediate upregulation of IFNγ, a different effect was observed against the other two cytokines.

In particular, while P2 was more efficient (compared to P1) in inhibiting the gliadin-mediated IL-15 upregulation and secretion [27] (Figure 3A,B, bottom panels, compare the two rightmost conditions, P1 and P2), P1 was the most efficient in reducing the gliadin-mediated IL-17A upregulation [28] (Figure 3A, middle panel, compare the two rightmost conditions, P1 and P2).

Collectively, our data confirm a prominent small intestinal inflammation induced by long-term gliadin exposure in our mouse model of gliadin sensitivity. Importantly, our data show that probiotics administration consistently mitigates the toxic effects of the active gliadin peptides, although to a different extent, possibly depending on the specific probiotic composition.

Importantly, the ability of probiotics in mitigating the proinflammatory and tissue damage activity of gliadin exposure was also evidenced by morphological analysis of small intestine section, as reported in Figure 3C. In fact, H/E staining of tissue from gliadin exposed mice showed a clear change in the morphology of villi (compatible with atrophy) compared to matched control. Combined gliadin and P1 or P2 treatment completely reverted the gliadin-mediated tissue damage (Figure 3C).

### 3.4. ER Stress Was Promptly Induced by Gliadin and Efficiently Inhibited by Probiotics Administration

**Figure 4:**
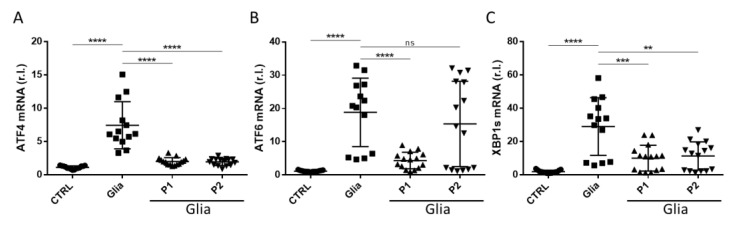
Endoplasmic reticulum (ER) Stress induced by gliadin exposure was buffered by probiotics. ATF4 (**A**), ATF6 (**B**), and XBP1s (**C**) expression levels were evaluated in the small intestine of Balb/c-fed third-generation gluten-free diet treated (Glia) on untreated (CTRL) with gliadin in the presence or absence of P1 or P2. Mean ± SD of triplicate sample; **** *p* < 0.0001; *** *p* < 0.001; ** *p* < 0.01; ns = not significant.

The ER is the site of synthesis and folding of lysosomal, membrane, and secretory proteins, which, collectively, represent a large fraction of the total protein output of a mammalian cell. The homeostasis of this compartment and, therefore, its function, is finely regulated by calcium concentration, redox potential, and the availability of chaperonins and cochaperonins. Extracellular or intracellular insults compromising the homeostasis of this organelle results in an impaired function, termed ER Stress, consisting of a luminal accumulation of misfolded proteins. In turn, ER Stress activates the so-called UPR [11]. The UPR function is primarily a pro-survival response aimed to restore the physiological functions of this compartment through the activation of a finely regulated genetic program. However, acute or unsustainable stress results in the activation of a UPR-mediated proapoptotic program [29,30]. Therefore, due to the key role played by ER and UPR in cell functions and stress management, it is not surprising that ER Stress (and UPR) has been implicated in the pathogenesis of many diseases and, in particular, in inflammatory disease, potentially contributing substantially to disease onset and progression [31]. In this context, Caputo and colleagues indicated the potential induction of ER Stress in vitro in Caco-2 cells exposed to gliadin [12]. To validate this hypothesis in vivo, we evaluated the activation of UPR in our mouse model of gluten sensitivity in animals exposed to gliadin for 6 weeks compared to unexposed controls. Data reported in Figure 4 clearly show a consistent upregulation of the three main ER Stress markers, ATF4, ATF6, and XBP1 (Figure 4), compatible with a chronic ER Stress condition.

Interestingly, ATF6 and XBP1 seemed to be the most upregulated compared to ATF4, potentially indicating that the degradation of misfolded proteins (ERAD) and upregulation of chaperonins/co-chaperonins are highly active compared to protein synthesis inhibition (PERK/eIF2α/ATF4 axis).

Importantly, the exposure of gliadin-treated mice to P1 or P2 completely abrogated the gliadin-mediate upregulation of both ATF4 and XBP1 (Figure 4A,C, respectively). Interestingly, while the expression of ATF6 was completely inhibited by P1, in the same experimental conditions, P2 failed since the levels of this factor remained elevated (Figure 4B, compare the two rightmost conditions). Further studies are required to define the role of elevated ATF6 in presence of gliadin and P2.

Therefore, our data indicate that ER Stress is promptly induced in gluten-sensitive mice upon gliadin exposure and that probiotics might be used to efficiently restore the homeostasis of this compartment, potentially buffering the gut inflammation. These findings could potentially be of interest in the context of the clinical treatment of patients with CD.

### 3.5. Probiotics Were Able to Efficiently Inhibit the Gliadin-Mediated ER Stress but Did Not Restore Physiological Levels of CFTR and TG2 In Vitro

**Figure 5:**
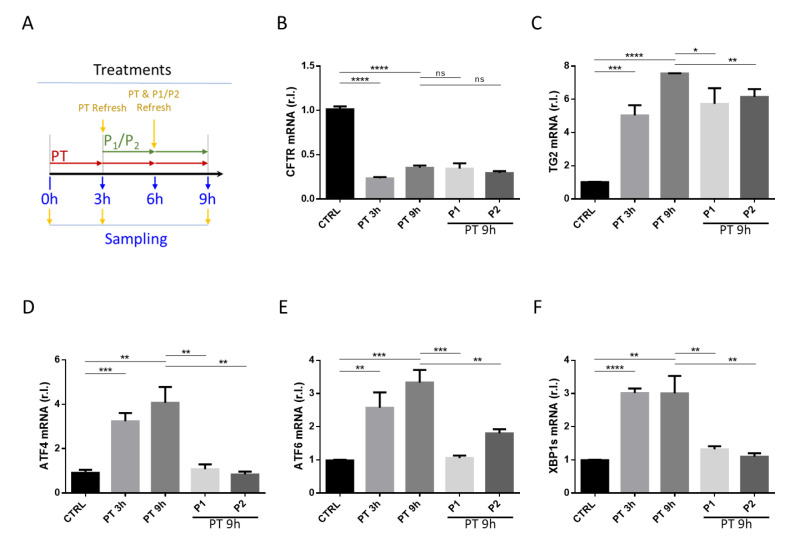
Impact of probiotics on peptide (PT)-mediated ER Stress induction in vitro. Caco-2 cells were untreated (CTRL) or treated with PT-gliadin for 3 (PT 3 h) or 9 (PT 9 h) hours in the presence or absence of P1 or P2 as schematically reported in panel (**A**). CFTR (**B**), TG2 (**C**), ATF4 (**D**), ATF6 (**E**), and XBP1s (**F**) expression levels were evaluated by qRT-PCR. Mean ± SD of triplicate sample; **** *p* < 0.0001; *** *p* < 0.001; ** *p* < 0.01; * *p* < 0.05; ns = not significant.

Finally, we tested the ability of the two probiotics formulations to restore the physiological ER homeostasis in vitro using Caco-2 cells as a model. To this aim, we exposed Caco-2 cells to pepsin-trypsin-gliadin digested peptides (PT) for 3 h and for more 6 h in the presence or absence of P1 or P2, while untreated cells were used as a control. Importantly, the medium containing PT ± P1 or P2 was replenished every 3 h, as reported in Figure 5A, since, after 3 h of treatment, it seems that PT loses its toxic activity (Supplementary Figure S1). Our data confirmed that PT efficiently downregulated the expression of CFTR as soon as 3 h, and its low expression level was stable at 9 h posttreatment (Figure 5B). In parallel, we observed a prompt upregulation of TG2 (at 3 h), which was still evident after 9 h of PT treatment (Figure 5C). In the same experimental conditions, we also confirmed the induction of UPR upon PT treatment at both 3 and 9 h (Figure 5D–F). Moreover, the presence of P1 or P2 failed in restoring the physiological expression levels of CFTR, at least at the mRNA level, confirming our in vivo data (Figure 1A).

In parallel, in contrast to in vivo data, both P1 and P2 were not able to inhibit the PT-mediated enhanced expression of TG (compare Figure 1B and Figure 5C), potentially indicating a microenvironment involvement in vivo.

Of note, both probiotics formulations efficiently and completely abrogated the PT-induced ER Stress, as evidenced by the complete inhibition of both ATF4 and XBP1 upregulation (Figure 5D,F). Importantly, although P1 also completely abrogated the PT-stimulated upregulation of ATF6, the expression levels of this factor were lower but still high in the presence of P2 (Figure 5E, compare the two rightmost histograms), confirming our in vivo data indicating a different activity of P1 and P2 on the gliadin-mediated altered expression of ATF6 (Figure 4 middle panel).

## 4. Discussion

Celiac Disease represents a global health problem, with a prevalence around 0.5–1% in the general population and approximately 1% of the population in the Western world [32,33]. CD is an autoimmune chronic disorder producing intestinal damages induced by gluten and gluten-related proteins, in which genetic susceptibility plays a key role. In fact, the consumption of gluten and/or gluten-related proteins by subjects with genetic predisposition represents the main environmental risk factor for CD. A major rule in the ignition of immune response toward gliadin peptides produced by partial enzymatic digestion in the intestine is played by tissue transglutaminase 2 (TG2). This enzyme is able to deaminate the glutamine rich gliadin-derived, resulting in an enhanced affinity to HLA-DQ2 or DQ8, finally resulting in downstream activation and CD4^+^T cell-mediated response [34]. Such response initiates a cascade, resulting in intestinal inflammation, villi atrophy, and enteropathy that can produce extended damage in the mucosa of the small intestine [35].

Although several clinical interventions have been proposed, the only globally accepted treatment for CD is a strict lifelong gluten-free diet (GFD). Although the majority of CD patients fully respond to GFD and have a normal life expectancy, elderly people, diagnostic delay, and poor adherence to GFD represent risk factors to develop disease complications such as refractory celiac disease (RCD), enteropathy-associated T-cell lymphoma (EATL), and small bowel carcinoma (SBC) [36,37,38].

The gastrointestinal (GI) tract is also a complex ecosystem in which the microbiota has been recently described as an ‘extra organ’ of the body. It is mainly constituted of bacteria, as well as archaea, viruses, protozoa, and fungi [39]. The adult human gastrointestinal tract harbors trillions of bacteria, including at least several hundred species and more than 6000 strains. However, this is not an isolated ecosystem but, on the contrary, it is intensely and actively connected with the host via a bidirectional intense communication. Indeed, it plays key roles in GI functions: Microbes facilitate the digestion and transformation of indigestible polysaccharides, provide vitamins, participate to the shaping of the intestinal epithelium, are involved in host immune defense against pathogens in the intestinal lumen, and contribute to the maintenance of intestinal homeostasis [39]. Although the community of the GI microbiota does not undergo significant fluctuations throughout adult life, antibiotic exposure, infections, lifestyle, and diet might profoundly affect it. Therefore, it is not surprising that altered microbiota homeostasis (dysbiosis) has been linked to the onset/progression of diseases characterized by inflammation of the GI tract, such as Crohn’s Disease, Ulcerative Colitis [40], and CD [41]. Importantly, the strong impact of microbiota on host health is not restricted to those pathologies but has also been evidenced in other human diseases ranging from cardiovascular, neurologic, respiratory, and metabolic illnesses to cancer [42]. However, the key question that arises with a not-yet-convincing answer is: Does dysbiosis precede or is it a consequence of disease?

Nonetheless, buffering the gut dysbiosis seems to offer a new treatment opportunity to mitigate, delay, or inhibit the progression of several human disorders. Indeed, in this context, diet supplementation with probiotics and prebiotics has been explored as a strategy to modulate the gut microbiome to an anti-inflammatory state. In line with these hypotheses, in the present study, we demonstrated that probiotics administration efficiently reduced the hallmarks of intestinal inflammation stimulated by gliadin in a mouse model of gluten sensitivity.

This work provides evidence of the occurrence of ER Stress in gliadin-induced intestinal inflammation in vivo (at least in our model) and that such cellular response can be mitigated by probiotics.

Importantly, this is the first evidence showing a gliadin-mediated TG2 and CFTR gene expression regulation, although the molecular mechanisms are still unclear. At this time, we can only speculate the potential involvement of components of the UPR, since ATF6 has been previously found to inhibit CFTR expression [43], while the PERK axis was found to positively modulate the expression of TG2 [44] under ER Stress conditions.

Finally, we found that while both probiotics formulation efficiently prevented the gliadin-mediated TG2 upregulation at both mRNA and protein levels, an apparent discrepancy was reported on CFTR at the mRNA and protein levels. In fact, while P1 and P2 did not affect the gliadin-mediated CFTR downregulation at the mRNA level, a partial recovery was observed at the protein level. Further studies are required to fully elucidate this result. However, we can hypothesize that probiotics might inhibit the degradation of CFTR protein stimulated by gliadin binding [5].

Importantly, our analysis indicated no major effects by GFD per se, since the basal expression of all evaluated markers was equal in mice fed with a standard vs. gluten free diet (Supplementary Figure S2).

Significantly, our data show that probiotics, although not interfering with the effects of active gliadin peptides on intestinal epithelial CFTR, have a beneficial effect, thus inhibiting gut inflammation, at least in a gluten-sensitive mice model. Moreover, our results also indicate a different effect of probiotics at the molecular level depending on the specific formulation. However, further studies are required to fully understand the molecular mechanisms by which probiotics inhibit the gliadin induced ER Stress and whether CD patients can also benefit from this treatment.

## Supplementary Materials

The following are available online at https://www.mdpi.com/article/10.3390/nu13041221/s1, Figure S1: Caco-2 treatment w/o refresh, Figure S2: Gliadin Free vs. Standard Diet, Table S1: Primers sequence.
