# Supplementary material for: Probiotics Supplements Reduce ER Stress and Gut Inflammation Associated with Gliadin Intake in a Mouse Model of Gluten Sensitivity
Source: Nutrients. 2021 Apr 7;13(4):1221. doi: 10.3390/nu13041221 (PMC8067866; doi:10.3390/nu13041221)
Supplement: Supplementary file 1 [file nutrients-13-01221-s001.pdf]

Supplementary Table1. Primers sequence.

Primer	Sequence
mIL-15_F	CAGCAAGGACCATGAAGA
mIL-15_R	GGCTGAGTTCCACATCTAAC
mIL-17a_F	CGCAATGAAGACCCTGATAG
mIL-17_R	CTTGCTGGATGAGAACAGAA
mINFγ_F	CCACATCTATGCCACTTGAG
mINFγ_R	CTCTTCCTCATGGCTGTTTC
mATF4_F	GTTTAGAGCTAGGCAGTGAAG
mATF4_R	CCTTTACACATGGAGGGATTAG
mATF6_F	GATGGTGACAACCAGAAAGA
mATF6_R	TGGAGGTGGAGGCATATAA
mXBP1s_F	AGTCCGCAGCAGGTG
mXBP1s_R	GGTCCAACTTGTCCAGAATG
mTG2_F	AAGAGCGAAGGGACATACT
mTG2_R	TGAGCACAGACCCATCTT
mCFRT_F	AGGAGGACAGGGATGATAAG
mCFTR_R	GTAGACACACCAGGAGTCTG
mCLD15_F	GGGACCCTCCACATACTT
mCLD15_R	CATACTTGGTTCCAGCATACA
mCLD2_F	CCTCGCTGGCTTGTATTATC
mCLD2_R	AAAGACTCCACCCACTACA
mOCLN_F	TCTTTGGAGGAAGCCTAAAC
mOCLN_R	CTGCTCTTGGGTCTGTATATC
mGAPDH_F	TTCAACGGCACAGTCAAG
mGAPDH_R	CCAGTAGACTCCACGACATA
hATF4_F	CCCGGAGAAGGCATCCTC
hATF4_R	GTGGCCAAGCACTTCAAACC
hATF6_F	TTTGCTGTCTCAGCCTACTGTGGT
hATF6_R	TCCATTCACTGGGCTATTCGCTGA
hXBP1s_F	AGAGAAAACTCATGGCCTTGTAGTTG
hXBP1s_R	CATTCCCCTTGGCTTCCG
hTG2_F	CCTGGCCTACGTGACGTGTC
hTG2_R	CGGCTTGGAGCTGGGTCT
hCFTR_F	CCACTGGTGCATACTCTAATC
hCFTR_R	CGTGTTGAGGGTTGACATAG
hL34_F	GTCCCGAACCCCTGGTAATAGA
hL34_R	GGCCCTGCTGACATGTTTCTT

Note*: m = mouse; h = human; XBP1s_F/R = primers designed to amplify the spliced (mature) mRNA of XBP1.*

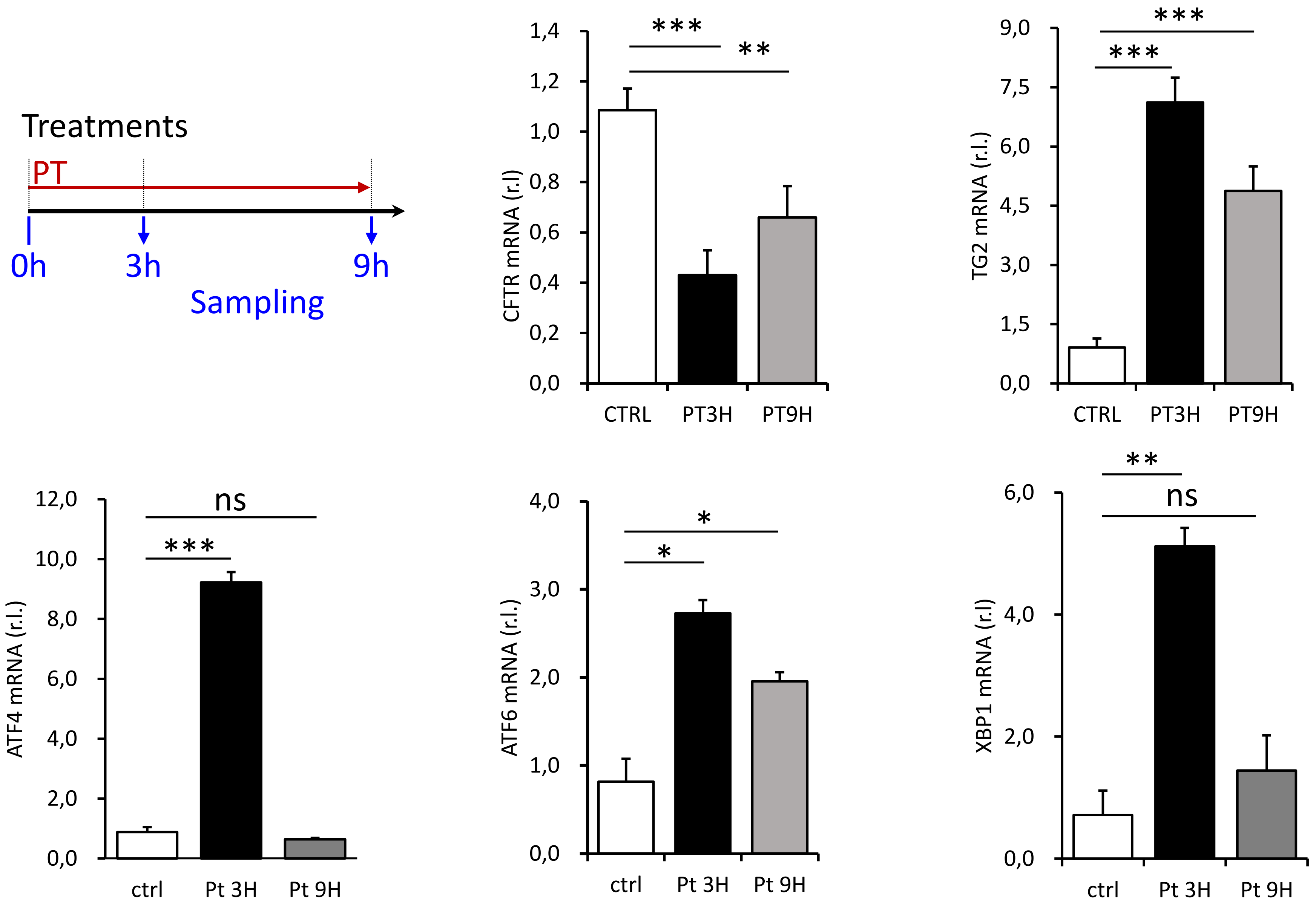

Supplementary Figure S1. Caco-2 tretament w/o refresh. Caco-2 cells were treated or untreated with PT and total RNA was extractet at indicate time points. The gene expression analysis of indicated markers was performed by qRT-PCR. Mean ± SD of triplicate sample, ns = differences not statistically significant (Student's t test).

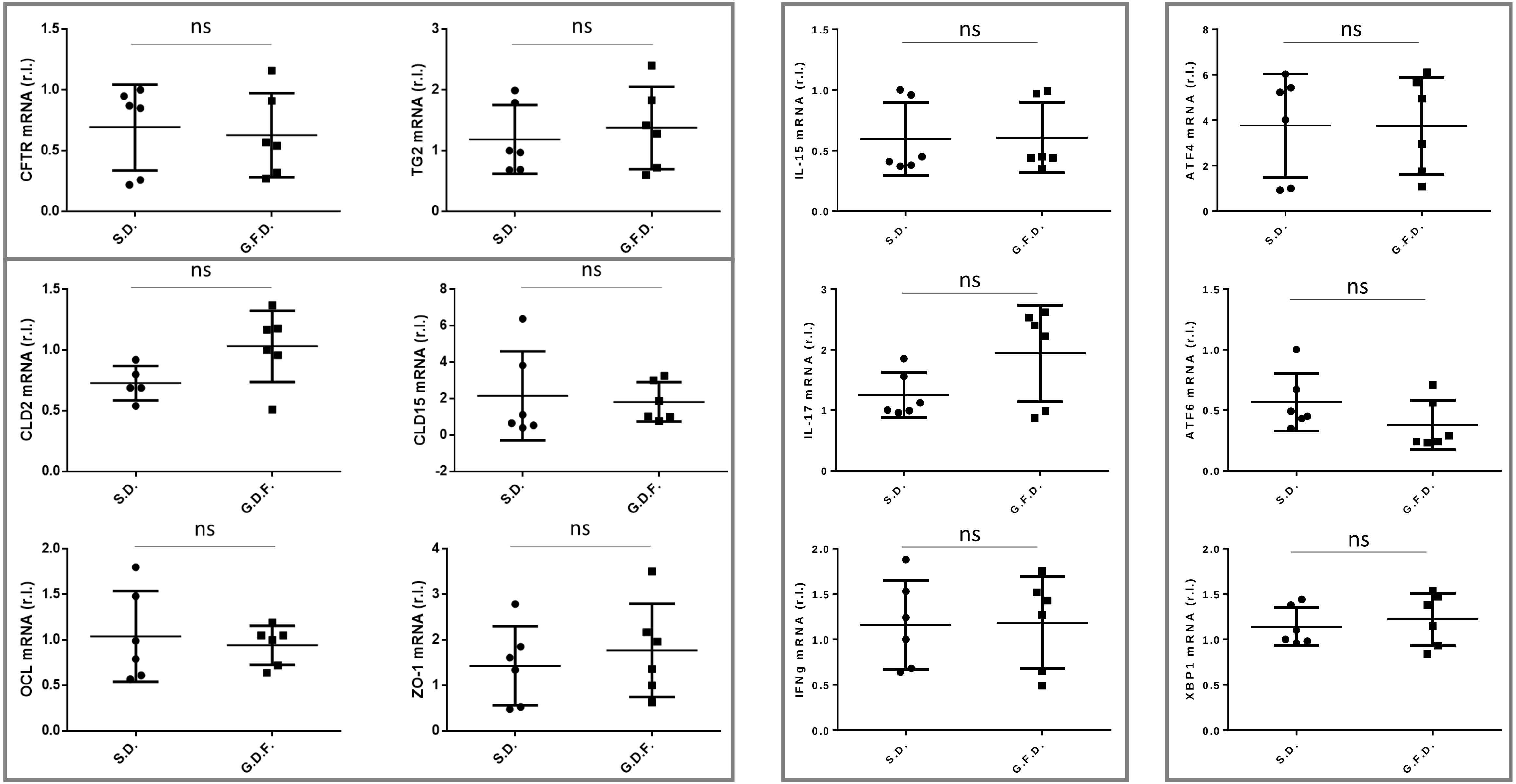

Supplementary Figure S2. Gliadin Free vs Standard Diet. The basal expression of indicated markers were analysed by qRT-PCR and compared between mice fed with a gliadin free diet (G.D.F.) and standard diet (S.D.). GAPDH was used as loading control. Mean ± SD of triplicate sample, ns = differences not statistically significant (Student's t test).
